# Neural Basis for the Ability of Atypical Antipsychotic Drugs to Improve Cognition in Schizophrenia

**DOI:** 10.3389/fnbeh.2013.00140

**Published:** 2013-10-16

**Authors:** Tomiki Sumiyoshi, Yuko Higuchi, Takashi Uehara

**Affiliations:** ^1^National Center of Neurology and Psychiatry, Kodaira, Tokyo, Japan; ^2^Department of Neuropsychiatry, University of Toyama Graduate School of Medicine and Pharmaceutical Sciences, Toyama, Japan

**Keywords:** atypical antipsychotics, second generation, cognitive function, 5-HT receptors, lactate, energy metabolism, neuropsychology, electrophysiology

## Abstract

Cognitive impairments are considered to largely affect functional outcome in patients with schizophrenia, other psychotic illnesses, or mood disorders. Specifically, there is much attention to the role of psychotropic compounds acting on serotonin (5-HT) receptors in ameliorating cognitive deficits of schizophrenia. It is noteworthy that atypical antipsychotic drugs (AAPDs), e.g., clozapine, melperone, risperidone, olanzapine, quetiapine, aripiprazole, perospirone, blonanserin, and lurasidone, have variable affinities for these receptors. Among the 5-HT receptor subtypes, the 5-HT_1A_ receptor is attracting particular interests as a potential target for enhancing cognition, based on preclinical and clinical evidence. The neural network underlying the ability of 5-HT_1A_ agonists to treat cognitive impairments of schizophrenia likely includes dopamine, glutamate, and gamma-aminobutyric acid neurons. A novel strategy for cognitive enhancement in psychosis may be benefited by focusing on energy metabolism in the brain. In this context, lactate plays a major role, and has been shown to protect neurons against oxidative and other stressors. In particular, our data indicate chronic treatment with tandospirone, a partial 5-HT_1A_ agonist, recover stress-induced lactate production in the prefrontal cortex of a rat model of schizophrenia. Recent advances of electrophysiological measures, e.g., event-related potentials, and their imaging have provided insights into facilitative effects on cognition of some AAPDs acting directly or indirectly on 5-HT_1A_ receptors. These findings are expected to promote the development of novel therapeutics for the improvement of functional outcome in people with schizophrenia.

## Introduction

Atypical antipsychotic drugs (AAPDs), sometimes called “second generation” antipsychotics, represent those exerting an antipsychotic efficacy at doses that do not cause extrapyramidal side effects (Meltzer, 1991, 2002; Sumiyoshi, 2008, 2013). With clozapine as the prototype, this class of agents includes risperidone, olanzapine, quetiapine, ziprasidone, aripiprazole, perospirone, blonanserin, paliperidone, iloperidone, asenapine, and lurasidone (Sumiyoshi, 2013). AAPDs share certain pharmacologic profiles in common, i.e., a relatively greater affinity for serotonin-5-HT_2A_ receptors relative to dopamine-D_2_ receptors (Meltzer et al., 1989; Stockmeier et al., 1993; Sumiyoshi et al., 1995). In contract, haloperidol, a typical antipsychotic drug (TAPD), shows a predominantly higher affinity for D_2_ receptors compared to other receptors (Meltzer et al., 1989; Stockmeier et al., 1993; Sumiyoshi et al., 1995). In addition to the higher 5-HT_2A_/D_2_ binding affinity ratio, there are some minor differences among the AAPDs. For example, perospirone and aripiprazole show a relatively greater affinity for 5-HT_1A_ receptors, while lurasidone demonstrates a relatively high affinity for 5-HT_7_ receptors (Sumiyoshi, 2013).

This paper provides a hypothesis regarding the neural basis for the ability of AAPDs to improve cognition. This theoretical issue is important from the perspective of the development of therapeutics for enhancing long-term outcome in patients with schizophrenia.

## Do AAPDs Enhance Cognition in Schizophrenia?

Typical antipsychotic drugs, such as perphenazine, have been reported to show some cognitive benefits in schizophrenia with a small effect size, as reported in the CATIE trial (Keefe et al., 2007). Importantly, Woodward et al. (2005) report an advantage of AAPDs over TAPDs in terms of enhancing cognition with a moderate effect size both in controlled and uncontrolled trials. However, there have been challenges to the pro-cognitive efficacy of AAPDs. For example, improvement of verbal memory by treatment with risperidone or olanzapine has been suggested to be no better than that of practice effect (or more precisely, test-retest effect) in normal controls (Goldberg et al., 2007). However, it may be premature to conclude that way, since no data were presented in that study (Goldberg et al., 2007) as to whether schizophrenia patients not receiving these AAPDs would have elicited the same degree of improvement as that in treated patients (Sumiyoshi, 2013).

One of the suggestions for this debate comes from the ability of lurasidone to dose-dependently improve cognitive functions, as measured by a computer-based test battery (Maruff et al., 2009), in a placebo-controlled double-blind study (Harvey et al., 2013; Sumiyoshi, 2013). This result provides a support for the ability of some AAPDs to enhance cognition in patients with schizophrenia, which is independent of a practice effect.

Another issue is what percentage of patients can be treated with a clinically meaningful degree. It is reported that a larger than 0.5 SD improvement in cognition substantially improves quality of life for patients (Norman et al., 2003). Accordingly, treatment with clozapine produced a significantly larger proportion of patients showing a larger than 0.5 SD improvement in letter fluency that predicts work outcome (Sumiyoshi and Meltzer, in preparation). Again, these findings provide a support for the proposition that AAPDs are superior over TAPDs for enhancing cognition.

In spite of these lines of evidence, cognitive benefits of AAPDs have been questioned, as noted above. One of the main reasons may be that the neural mechanisms for it have not been fully elucidated. Therefore, the following sections address this issue from the perspective of electrophysiological imaging, neural network, and energy metabolism.

## Electrophysiological Imaging

**Figure 1:**
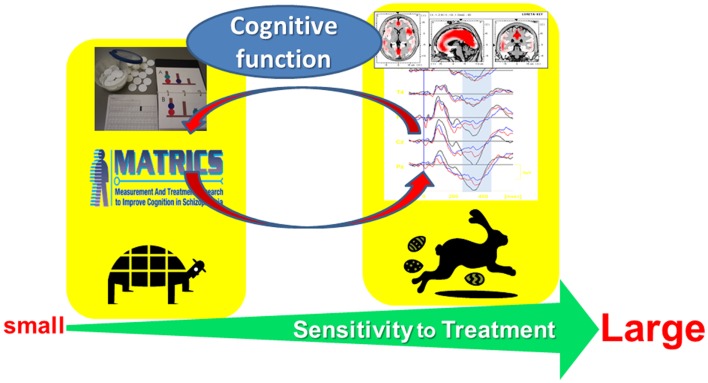
**Electrophysiological approach toward cognitive assessment in schizophrenia**. This strategy is expected to facilitate (1) elucidation of mechanisms of cognitive enhancement, (2) rational choice of psychotropic drugs, and (3) prediction of functional outcome.

Figure 1 illustrates a rationale for electrophysiological approach toward cognitive assessment. The combination of neuropsychological and electrophysiological methods, e.g., event-related potentials (ERPs), may be beneficial for the understanding of mechanisms of cognitive enhancement, rational choice of psychotropic drugs, and prediction of functional outcome.

**Figure 2:**
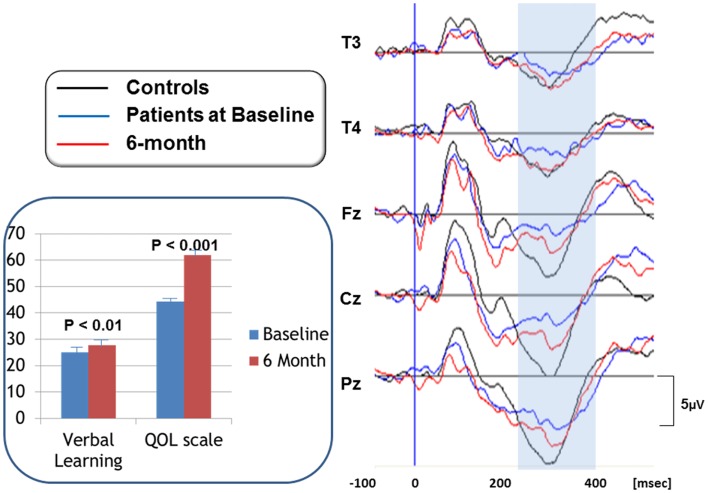
**P300 waveforms in response to olanzapine treatment (right)**. P300 waveforms were averaged for 16 subjects per group. Black lines represent P300 activity for control subjects. Blue lines show P300 for patients at baseline, whose amplitudes are diminished compared to control subjects. After 6 month treatment with olanzapine, P300 amplitudes were increased, as indicated by redlines. Scores of verbal memory and quality of life were also increased by olanzapine (inset). Bars represent mean + SE.

Specifically, we reported the effect of olanzapine on cognition and QOL, as well as P300, a component of ERPs, in patients with schizophrenia (Higuchi et al., 2008). P300 has been used as a marker of attentive cognitive processes. Figure 2 (right) demonstrates P300 waveforms. At baseline, P300 amplitudes of patients were diminished compared to those of control subjects. After 6 month treatment with olanzapine, P300 amplitudes were increased, as were scores of verbal memory and quality of life (Figure 2, left).

**Figure 3:**
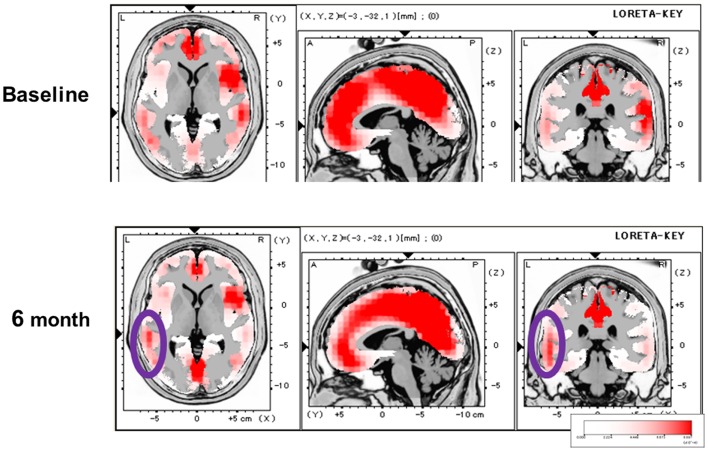
**Effect of olanzapine on P300 current density, evaluated by the LORETA methods, in patients with schizophrenia**. At baseline, P300 current density in the left superior temporal gyrus was decreased. Olanzapine increased P300 current density in this brain region (circled), and this pattern of three-dimensional configuration was similar to that in control subjects.

We subsequently evaluated the effect of olanzapine on P300 current source density in discrete brain areas (Higuchi et al., 2008) (Figure 3). At baseline, P300 current density in the left superior temporal gyrus (STG) was decreased in patients. Olanzapine increased P300 current density in the left STG, but not other regions, such as the prefrontal cortex (PFC). In fact, this left-dominant pattern of P300 current density is similar to that for control subjects. These observations provide the first evidence that AAPDs ameliorate neurocognitive disturbances by correcting three-dimensional distribution of electrophysiological activity (Sumiyoshi et al., 2006, 2009; Higuchi et al., 2008).

**Figure 4:**
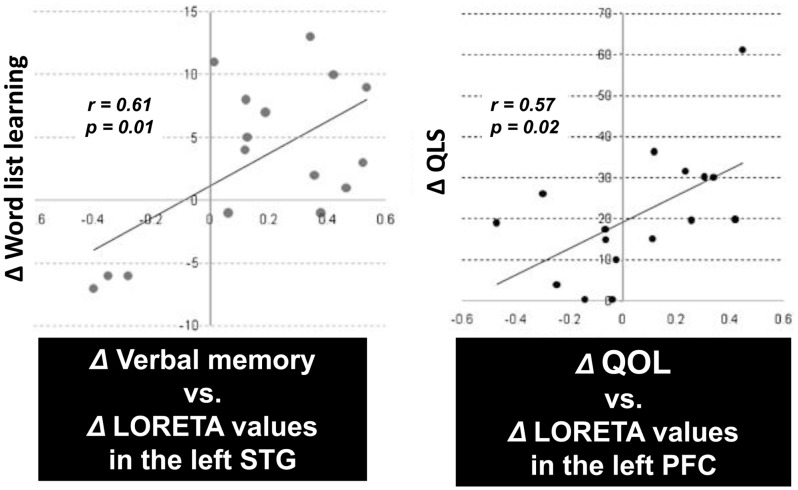
**P300 current density change vs. cognition/QOL changes in patients treated with olanzapine**. There was a significant positive correlation between improvement of verbal memory and enhancement of P300 current density in the left superior temporal gyrus (STG) (*left*). Also, the improvement of the Quality of Life score was correlated with enhancement of P300 current density in the left prefrontal cortex (PFC) (*right*).

An important aspect of this study was the correlation between the change in P300 current density and cognition or functional outcome. In fact, there was a significant positive correlation between improvement of verbal memory and enhancement of P300 current density in the left STG (Figure 4, right). Also, the change in the Quality of Life score (Heinrichs et al., 1984) was significantly correlated with enhancement of P300 current density in the left PFC (Figure 4, left). These results indicate that the change of regional electrophysiological activities in response to treatment can predict enhancement of cognitive and functional outcomes (Higuchi et al., 2008; Sumiyoshi et al., 2009, 2011).

**Figure 5:**
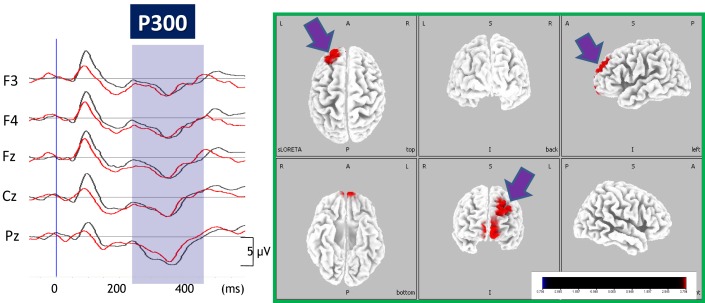
**Enhancement of P300 current density by perospirone in schizophrenia**. *Left*, grand average of ERP waveforms before (black lines) and after (red lines) treatment with perospirone in patients with schizophrenia. *Right*, 6 months treatment with perospirone enhanced P300 current density, evaluated by the sLORETA method, in the left superior frontal gyrus. Perospirone also improved social cognition, the degree of which was correlated with P300 activity in the frontal brain regions (see text).

We also investigated the effect of perospirone on P300 current density (Sumiyoshi et al., 2009). Perospirone is one of the AAPDs marketed in Japan, and has high affinity for 5-HT_1A_ receptors (Araki et al., 2006; Sumiyoshi et al., 2009; Higuchi et al., 2013). Unlike the case for olanzapine, perospirone enhanced P300 current density in the left PFC in patients with schizophrenia (Figure 5). This change was correlated with improvement of cognitive function relevant to daily living skills (Sumiyoshi et al., 2009).

These observations are consistent with our previous report that 5-HT_1A_ receptor density is increased in the left PFC from subjects with schizophrenia (Sumiyoshi et al., 1996). The up-regulation of 5-HT_1A_ receptors is hypothesized to reflect a compensatory reaction to diminished neurotransmission through these receptors (Sumiyoshi et al., 1996). The electrophysiological findings, mentioned here, may be consistent with this hypothesis, and explain distinct cognition-enhancing profiles of some AAPDs with high affinity for 5-HT_1A_ receptors, e.g., ziprasidone, perospirone, aripiprazole, and lurasidone (Sumiyoshi, 2012, 2013, in press; Sumiyoshi and Higuchi, 2013). This concept may explain why perospirone, but not olanzapine enhanced P300 current density in the PFC.

## Neural Network Mediating Cognitive Enhancement of AAPDs

**Figure 6:**
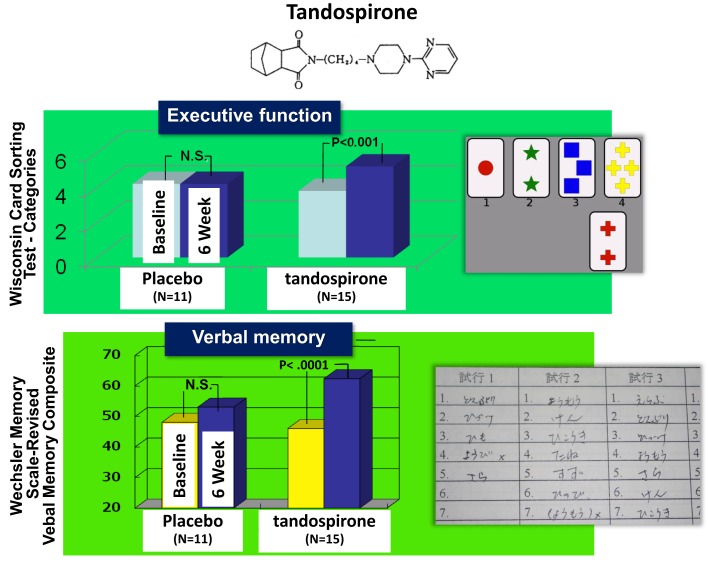
**Effect of tandospirone, a 5-HT_1A_ partial agonist, on cognition in schizophrenia**. Six-week treatment with tandospirone, but not placebo enhanced executive function (effect size = 0.63) and verbal memory (0.70), two cognitive domains relevant to functional outcome, in patients receiving haloperidol.

As discussed, 5-HT_1A_ receptor agonism has been suggested to enhance cognition [see also Sumiyoshi et al. (2008)]. In fact, the addition of tandospirone, a 5-HT_1A_ partial agonist, improved executive function and verbal memory in patients treated with TAPDs (Sumiyoshi et al., 2001a,b) (Figure 6). Data from these clinical trials suggest 5-HT_1A_ agonists enhance some of the key cognitive domains, including those associated with frontal cortical function.

**Figure 7:**
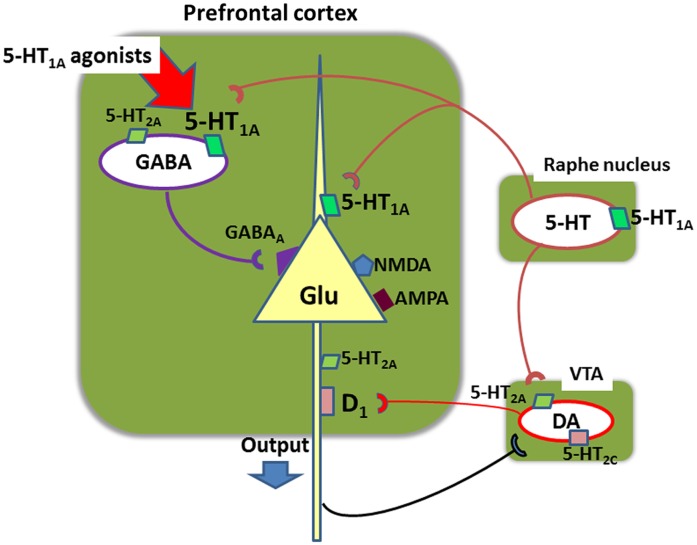
**Neural network in the prefrontal cortex involving glutamate (Glu), gamma-aminobutyric acid (GABA), serotonin (5-HT), and dopamine (DA) neurons**. Systemic administration of 5-HT_1A_ agonists, such as 8-OH-DPAT, inhibits action potentials of GABA neurons, leading to disinhibition of glutamate neurons (Llado-Pelfort et al., 2011; Sumiyoshi and Higuchi, 2013). This also leads to activation of meso-cortical dopamine neurons. For example, administration of clozapine, a 5-HT_1A_ agonist, increases extracellular DA concentrations in the prefrontal cortex in mice, but it does not occur in mutant mice lacking 5-HT_1A_ receptors (Bortolozzi et al., 2010). These neural events may explain the ability of augmentation therapy with tandospirone to restore mismatch negativity amplitudes (Higuchi et al., 2010), an electrophysiological measure of glutamatergic activity that is diminished in schizophrenia. VTA, ventral tegmental area.

Figure 7 illustrates a neural network providing a possible basis for the ability of tandospirone and AAPDs acting on 5-HT_1A_ receptors to enhance cognition. Systemic administration of 5-HT_1A_ agonists has been shown to selectively stimulate 5-HT_1A_ receptors located on gamma-aminobutyric acid (GABA) interneurons in the PFC (Llado-Pelfort et al., 2011; Sumiyoshi and Higuchi, 2013). This diminishes the activity of GABA neurons, leading to disinhibition of Glu neurons. This may explain the ability of AAPDs to augment DA release in the PFC (Sumiyoshi and Higuchi, 2013), a putative mechanism for the ability of AAPDs to enhance cognition, in a 5-HT_1A_ receptor-dependent manner (Diaz-Mataix et al., 2005; Bortolozzi et al., 2010). These neural events may explain the ability of augmentation therapy with tandospirone to restore mismatch negativity amplitudes (Higuchi et al., 2010), an electrophysiological measure of glutamatergic activity that is diminished in schizophrenia (Javitt et al., 2008). Other possible mechanisms may involve GABA_B_ receptor-mediated transmissions (Gronier, 2008) or other neurotransmitters (e.g., acetylcholine).

## Role for Energy Metabolism

Traditionally, energy supply into the brain has been considered to depend on glucose. However, recent research suggests lactate plays a significant role in energy production both in the aerobic and anaerobic conditions, irrespective of the presence of glucose (Wyss et al., 2011; Uehara and Sumiyoshi, 2013).

The lactate-dependent energy metabolism has been associated with glutamatergic activity (Uehara et al., 2008). Specifically, glutamatergic transmissions enhance lactate production, which is mediated by *N*-methyl-d-aspartate (NMDA) receptors and glutamate transporters, as well as astrocytes (Uehara et al., 2008; Uehara and Sumiyoshi, 2013). Recently, lactate has been shown to exert neuroprotective effects (Wyss et al., 2011). These lines of evidence prompted us to use lactate metabolism as a biological basis for the effect of pro-cognitive drugs.

**Figure 8:**
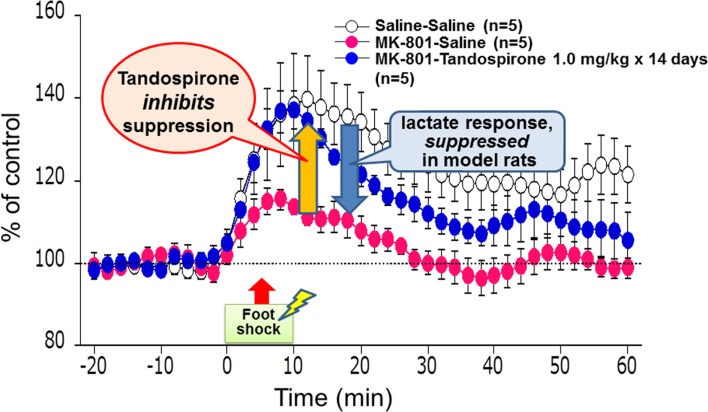
**Effect of tandospirone on lactate production in the prefrontal cortex of a rat model of schizophrenia, as measured by *in vivo* microdialysis**. At the neonatal stage, rats were transiently injected with MK-801, an antagonist at the *N*-methyl-d-aspartate receptor, on postnatal days 7–10. These model animals showed suppression of the stress-induced increment of lactate levels, as represented by extracellular concentrations, in the adult stage. This suppression in model rats was inhibited by chronic treatment with tandospirone (once daily for 14 days before the measurement of lactate levels).

Measurement of lactate in the extracellular space can provide real-time information on its production (Uehara et al., 2008). Lactate metabolism was hypothesized to reflect energy supply in the brain areas crucial for cognitive functions, e.g., PFC. Figure 8 describes the effect of tandospirone on extracellular lactate concentrations in a rat model of schizophrenia (Uehara et al., 2012). At the neonatal stage (postnatal days 7–10), rats were transiently administered MK-801, an antagonist at the NMDA receptor. In this experiment, these model rats showed suppression of the stress-induced increment of lactate levels in the PFC, suggesting impaired energy metabolism. This suppression in the model rats was inhibited by chronic treatment with tandospirone (once daily for 14 days before the measurement of lactate levels) (Uehara et al., 2012). These results are consistent with clinical observations that 5-HT_1A_ agonists, such as tandospirone and buspirone, ameliorate cognitive impairment related to PFC function (Sumiyoshi et al., 2001a,b, 2007).

## Perspectives

A main topic of this article has been the role for 5-HT_1A_ receptors in cognitive improvement. On the other hand, other 5-HT receptor subtypes have been suggested to be a potential candidate for cognitive enhancers. These include 5-HT_3_ (e.g., mirtazapine, ondansetron), 5-HT_6_ (Ro04-06790, Lu AE58054), and 5-HT_7_ (SB25874, amisulpride, lurasidone) receptors [reviewed in Sumiyoshi and Higuchi (2013)].

Another issue to be considered in the development of promising agents is the assessment of functional outcome, in addition to neurocognition (neuropsychological performance, or “primary measures”). In this context, intermediate functional measures, or “co-primary measures,” have attracted interest as a target for therapeutic intervention (Sumiyoshi and Sumiyoshi, in press). For example, a greater sensitivity to treatment has been reported for co-primary measures compared to primary measures in a clinical trial of lurasidone and ziprasidone (Harvey et al., 2011). Therefore, intermediate functional measures (co-primary measures) deserve more attention in the development of novel pharmacotherapy for schizophrenia and related illnesses.

In conclusion, AAPDs have been shown to enhance cognition in a clinically meaningful manner. The mechanisms for it may include several modes of action and neural networks, which requires further explorations.

## Conflict of Interest Statement

The authors declare that the research was conducted in the absence of any commercial or financial relationships that could be construed as a potential conflict of interest.

## References

[B1] Araki T. Yamasue H. Sumiyoshi T. Kuwabara H. Suga M. Iwanami A. (2006). Perospirone in the treatment of schizophrenia: effect on verbal memory organization. Prog. Neuropsychopharmacol. Biol. Psychiatry 30, 204–208 10.1016/j.pnpbp.2005.10.015 16300872

[B2] Bortolozzi A. Masana M. Diaz-Mataix L. Cortes R. Scorza M. C. Gingrich J. A. (2010). Dopamine release induced by atypical antipsychotics in prefrontal cortex requires 5-HT1A receptors but not 5-HT2A receptors. Int. J. Neuropsychopharmacol. 13, 1299–1314 10.1017/S146114571000009X 20158933 PMC6112770

[B3] Diaz-Mataix L. Scorza M. C. Bortolozzi A. Toth M. Celada P. Artigas F. (2005). Involvement of 5-HT1A receptors in prefrontal cortex in the modulation of dopaminergic activity: role in atypical antipsychotic action. J. Neurosci. 25, 10831–10843 10.1523/JNEUROSCI.2999-05.2005 16306396 PMC6725886

[B4] Goldberg T. E. Goldman R. S. Burdick K. E. Malhotra A. K. Lencz T. Patel R. C. (2007). Cognitive improvement after treatment with second-generation antipsychotic medications in first-episode schizophrenia: is it a practice effect? Arch. Gen. Psychiatry 64, 1115–1122 10.1001/archpsyc.64.10.1115 17909123

[B5] Gronier B. (2008). Involvement of glutamate neurotransmission and *N*-methyl-d-aspartate receptor in the activation of midbrain dopamine neurons by 5-HT_1A_ receptors agonists: an electrophysiological study in the rat. Neuroscience 156, 995–1004 10.1016/j.neuroscience.2008.08.033 18801415

[B6] Harvey P. D. Ogasa M. Cucchiaro J. Loebel A. Keefe R. S. (2011). Performance and interview-based assessments of cognitive change in a randomized, double-blind comparison of lurasidone vs. ziprasidone. Schizophr. Res. 127, 188–194 10.1016/j.schres.2011.01.004 21277745

[B7] Harvey P. D. Siu C. O. Hsu J. Cucchiaro J. Maruff P. Loebel A. (2013). Effect of lurasidone on neurocognitive performance in patients with schizophrenia: a short-term placebo- and active-controlled study followed by a 6-month double-blind extension. Eur. Neuropsychopharmacol. 10.1016/j.euroneuro.2013.08.003 24035633

[B8] Heinrichs D. W. Hanlon T. E. Carpenter W. T. J. (1984). The quality of life scale: an instrument for rating the schizophrenic deficit syndrome. Schizophr. Bull. 10, 388–398 10.1093/schbul/10.3.388 6474101

[B9] Higuchi Y. Sumiyoshi T. Ito T. Suzuki M. (2013). Perospirone normalized P300 and cognitive function in a case of early psychosis. J. Clin. Psychopharmacol. 33, 263–266 10.1097/JCP.0b013e318287c527 23422402

[B10] Higuchi Y. Sumiyoshi T. Kawasaki Y. Ito T. Seo T. Suzuki M. (2010). Effect of tandospirone on mismatch negativity and cognitive performance in schizophrenia: a case report. J. Clin. Psychopharmacol. 30, 732–734 10.1097/JCP.0b013e3181faa57d 21057236

[B11] Higuchi Y. Sumiyoshi T. Kawasaki Y. Matsui M. Arai H. Kurachi M. (2008). Electrophysiological basis for the ability of olanzapine to improve verbal memory and functional outcome in patients with schizophrenia: a LORETA analysis of P300. Schizophr. Res. 101, 320–330 10.1016/j.schres.2008.01.020 18321680

[B12] Javitt D. C. Spencer K. M. Thaker G. K. Winterer G. Hajos M. (2008). Neurophysiological biomarkers for drug development in schizophrenia. Nat. Rev. Drug Discov. 7, 68–83 10.1038/nrd2463 18064038 PMC2753449

[B13] Keefe R. S. Bilder R. M. Davis S. M. Harvey P. D. Palmer B. W. Gold J. M. (2007). Neurocognitive effects of antipsychotic medications in patients with chronic schizophrenia in the CATIE trial. Arch. Gen. Psychiatry 64, 633–647 10.1001/archpsyc.64.6.633 17548746

[B14] Llado-Pelfort L. Santana N. Ghisi V. Artigas F. Celada P. (2011). 5-HT1A receptor agonists enhance pyramidal cell firing in prefrontal cortex through a preferential action on GABA interneurons. Cereb. Cortex 22, 1487–1497 10.1093/cercor/bhr220 21893679

[B15] Maruff P. Thomas E. Cysique L. Brew B. Collie A. Snyder P. (2009). Validity of the CogState brief battery: relationship to standardized tests and sensitivity to cognitive impairment in mild traumatic brain injury, schizophrenia, and AIDS dementia complex. Arch. Clin. Neuropsychol. 24, 165–178 10.1093/arclin/acp010 19395350

[B16] Meltzer H. Y. (1991). The mechanism of action of novel antipsychotic drugs. Schizophr. Bull. 17, 263–287 10.1093/schbul/17.2.263 1679253

[B17] Meltzer H. Y. (2002). Action of atypical antipsychotics. Am. J. Psychiatry 159, 153–154 10.1176/appi.ajp.159.1.153-a 11772718

[B18] Meltzer H. Y. Matsubara S. Lee J. C. (1989). Classification of typical and atypical antipsychotic drugs on the basis of dopamine D-1, D-2 and serotonin2 pKi values. J. Pharmacol. Exp. Ther. 251, 238–246 2571717

[B19] Norman G. R. Sloan J. A. Wyrwich K. W. (2003). Interpretation of changes in health-related quality of life. Med. Care 41, 582–592 10.1097/00005650-200305000-00004 12719681

[B20] Stockmeier C. A. Dicarlo J. J. Zhang Y. Thompson P. Meltzer H. Y. (1993). Characterization of typical and atypical antipsychotic drugs based on in vivo occupancy of serotonin_2_ and dopamine_2_ receptors. J. Pharmacol. Exp. Ther. 266, 1374–1384 8103793

[B21] Sumiyoshi C. Sumiyoshi T. (in press). Functional outcome in patients with schizophrenia: the concept and the measurement. Act Nerv. Super.7914044

[B22] Sumiyoshi T. (2008). A possible dose-side effect relationship of antipsychotic drugs: Relevance to cognitive function in schizophrenia. Expert. Rev. Clin. Pharmacol. 1, 791–802 10.1586/17512433.1.6.791 24410608

[B23] Sumiyoshi T. (2012). Serotonin 1A receptors in the action of antipsychotic drugs. J. Psychopharmacol. 26, 1283–1284 10.1177/0269881112449398 22854647

[B24] Sumiyoshi T. (2013). Antipsychotic treatments; Focus on lurasidone. Front. Psychopharmacol. 26:102 10.3389/fphar.2013.00102 PMC3753015 23986702

[B25] Sumiyoshi T. (in press). Serotonin 5-HT1A receptors in the action of aripiprazole. J. Clin. Psychopharmacol. 10.1097/JCP.0000000000000135 24717255

[B26] Sumiyoshi T. Bubenikova-Valesova V. Horacek J. Bert B. (2008). Serotonin1A receptors in the pathophysiology of schizophrenia: development of novel cognition-enhancing therapeutics. Adv. Ther. 25, 1037–1056 10.1007/s12325-008-0102-2 18839076

[B27] Sumiyoshi T. Higuchi Y. (2013). Facilitative effect of serotonin1A receptor agonists on cognition in patients with schizophrenia. Curr. Med. Chem. 20, 357–362 10.2174/092986713804870846 23157627

[B28] Sumiyoshi T. Higuchi Y. Itoh T. Kawasaki Y. (2011). “Electrophysiological imaging evaluation of schizophrenia and treatment response,” in Handbook of Schizophrenia Spectrum Disorders, ed. Risner M. S. (New York: Springer).

[B29] Sumiyoshi T. Higuchi Y. Itoh T. Matsui M. Arai H. Suzuki M. (2009). Effect of perospirone on P300 electrophysiological activity and social cognition in schizophrenia: a three-dimensional analysis with sLORETA. Psychiatry Res. 172, 180–183 10.1016/j.pscychresns.2008.07.005 19386475

[B30] Sumiyoshi T. Higuchi Y. Kawasaki Y. Matsui M. Kato K. Yuuki H. (2006). Electrical brain activity and response to olanzapine in schizophrenia: a study with LORETA images of P300. Prog. Neuropsychopharmacol. Biol. Psychiatry 30, 1299–1303 10.1016/j.pnpbp.2006.04.028 16769169

[B31] Sumiyoshi T. Matsui M. Nohara S. Yamashita I. Kurachi M. Sumiyoshi C. (2001a). Enhancement of cognitive performance in schizophrenia by addition of tandospirone to neuroleptic treatment. Am. J. Psychiatry 158, 1722–1725 10.1176/appi.ajp.158.10.1722 11579010

[B32] Sumiyoshi T. Matsui M. Yamashita I. Nohara S. Kurachi M. Uehara T. (2001b). The effect of tandospirone, a serotonin(1A) agonist, on memory function in schizophrenia. Biol. Psychiatry 49, 861–868 10.1016/S0006-3223(00)01025-8 11343682

[B33] Sumiyoshi T. Park S. Jayathilake K. Roy A. Ertugrul A. Meltzer H. Y. (2007). Effect of buspirone, a serotonin1A partial agonist, on cognitive function in schizophrenia: a randomized, double-blind, placebo-controlled study. Schizophr. Res. 95, 158–168 10.1016/j.schres.2007.06.008 17628435

[B34] Sumiyoshi T. Stockmeier C. A. Overholser J. C. Dilley G. E. Meltzer H. Y. (1996). Serotonin_1A_ receptors are increased in postmortem prefrontal cortex in schizophrenia. Brain Res. 708, 209–214 10.1016/0006-8993(95)01361-X 8720882

[B35] Sumiyoshi T. Suzuki K. Sakamoto H. Yamaguchi N. Mori H. Shiba K. (1995). Atypicality of several antipsychotics on the basis of in vivo dopamine-D2 and serotonin-5HT2 receptor occupancy. Neuropsychopharmacology 12, 57–64 10.1038/sj.npp.1380239 7766287

[B36] Uehara T. Itoh H. Matsuoka T. Rujescu D. Genius J. Seo T. (2012). Effect of transient blockade of *N*-methyl-d-aspartate receptors at neonatal stage on stress-induced lactate metabolism in the medial prefrontal cortex of adult rats: role of 5-HT1A receptor agonism. Synapse 66, 408–417 10.1002/syn.21529 22213269

[B37] Uehara T. Sumiyoshi T. (2013). “Lactate metabolism as a new target for the therapeutics of schizophrenia,” in Frontiers in Clinical Drug Research-CNS and Neurological Disorders, ed. Rahman A. U. (Sharjah: Bentham Science Publishers).

[B38] Uehara T. Sumiyoshi T. Itoh H. Kurata K. (2008). Lactate production and neurotransmitters; evidence from microdialysis studies. Pharmacol. Biochem. Behav. 90, 273–281 10.1016/j.pbb.2008.04.001 18502489

[B39] Woodward N. D. Purdon S. E. Meltzer H. Y. Zald D. H. (2005). A meta-analysis of neuropsychological change to clozapine, olanzapine, quetiapine, and risperidone in schizophrenia. Int. J. Neuropsychopharmacol. 8, 457–472 10.1017/S146114570500516X 15784157

[B40] Wyss M. T. Jolivet R. Buck A. Magistretti P. J. Weber B. (2011). In vivo evidence for lactate as a neuronal energy source. J. Neurosci. 31, 7477–7485 10.1523/JNEUROSCI.0415-11.2011 21593331 PMC6622597

